# Fever management with or without a temperature control device after out‐of‐hospital cardiac arrest and resuscitation (TEMP‐CARE): A study protocol for a randomized clinical trial

**DOI:** 10.1111/aas.70034

**Published:** 2025-04-13

**Authors:** Johan Holgersson, V. Niemelä, M. B. Skrifvars, C. Kamp‐Jorgensen, M. Saxena, P. Young, F. Bass, J. Dankiewicz, N. Hammond, J. Hästbacka, H. Levin, G. Lilja, M. Moseby‐Knappe, M. Tiainen, M. Reinikainen, A. Ceric, J. Düring, A. Lybeck, D. Rodriguez‐Santos, J. Johnsson, J. Unden, A. Lundin, J. Kåhlin, J. Grip, J. Rosell, E. M. Lotman, L. Navarra, B. Crichton, D. Knight, A. Williams, L. Romundstad, P. Seidel, P. Stammet, T. Graf, A. Mengel, C. Leithner, J. Nee, P. Druwé, K. Ameloot, M. Wise, J. Riddel, M. Ahmed, M. Buckel, P. Mc Guigan, R. Maharaj, D. Wyncoll, M. Thomas, J. White, T. R. Keeble, D. Pogson, A. Nichol, M. Haenggi, M. P. Hilty, M. Iten, C. Schrag, M. Nafi, M. Joannidis, C. Robba, T. Pellis, J. Belohlavek, O. Smid, D. Rob, Y. Arabi, S. Buabbas, C. Yew Woon, A. Aneman, A. Stewart, S. Bernard, C. Palmer‐Simpson, N. Simpson, M. Ramanan, M. Reade, A. Delaney, B. Venkatesh, J. Tirkkonen, T. Oksanen, T. Kaakinen, S. Bendel, H. Friberg, T. Cronberg, J. Jakobsen, N. Nielsen

**Affiliations:** ^1^ Department of Clinical Sciences Lund, Anesthesia and Intensive Care Lund University Lund Sweden; ^2^ Department of Anesthesia and Intensive Care Helsingborg Hospital Helsingborg Sweden; ^3^ Department of Anaesthesia and Intensive Care Helsinki University Hospital and University of Helsinki Helsinki Finland; ^4^ Copenhagen Trial Unit, Centre for Clinical Intervention Research, The Capital Region Copenhagen University Hospital – Rigshospitalet Copenhagen Denmark; ^5^ Department of Regional Health Research, The Faculty of Health Sciences University of Southern Denmark Odense Denmark; ^6^ Critical Care Division and Department of Intensive Care Medicine, The George Institute for Global Health and St George Hospital Clinical School University of New South Wales Sydney Australia; ^7^ Intensive Care Unit, Wellington Hospital Wellington New Zealand; ^8^ Medical Research Institute of New Zealand Wellington New Zealand; ^9^ Australian and New Zealand Intensive Care Research Centre Monash University Melbourne Victoria Australia; ^10^ Department of Critical Care University of Melbourne Melbourne Victoria Australia; ^11^ Critical Care Program The George Institute for Global Health Sydney Australia; ^12^ Royal North Shore Hospital Sydney Australia; ^13^ Department of Clinical Sciences Lund, Section of Cardiology Skåne University Hospital Malmö Sweden; ^14^ Critical Care Program The George Institute for Global Health, UNSW Sydney Australia; ^15^ Wellbeing Services County of Pirkanmaa and Tampere University, Faculty of Medicine and Health Technology Tampere University Hospital Tampere Finland; ^16^ Department of Clinical Sciences Lund Lund University Lund Sweden; ^17^ Department of Research, Development, Education and Innovation Skåne University Hospital Lund Sweden; ^18^ Department of Neurology, Department of Clinical Sciences Lund Lund University Lund Sweden; ^19^ Department of Neurology Skåne University Hospital Lund Sweden; ^20^ Department of Neurology and Rehabilitation Skåne University Hospital Lund Sweden; ^21^ Department of Neurology Helsinki University Hospital and University of Helsinki Finland; ^22^ University of Eastern Finland Institute of Clinical Medicine; ^23^ Department of Anaesthesiology and Intensive Care Kuopio University Hospital; ^24^ Anesthesia and Intensive Care, Department of Clinical Sciences, Lund University Skane University Hospital Malmö Sweden; ^25^ Anesthesia and Intensive Care, Department of Clinical Sciences Lund, Lund University Skane University Hospital Lund Sweden; ^26^ Department of Anaesthesia and Intensive Care Skaraborgs Sjukhus Skövde Skaraborg Sweden; ^27^ Department of Anaesthesiology and Intensive Care Helsingborg Hospital Helsingborg Sweden; ^28^ Department of Anaesthesiology and Intensive Care Hallands Hospital Halmstad Halmstad Sweden; ^29^ Department of Intensive and Perioperative Care Skåne University Hospital, Lund University Lund Sweden; ^30^ Department of Anaesthesiology and Intensive Care Medicine Institute of Clinical Sciences, Sahlgrenska Academy, University of Gothenburg Gothenburg Sweden; ^31^ Perioperative Medicine and Intensive Care (PMI) Karolinska University Hospital Stockholm Sweden; ^32^ Department of Physiology and Pharmacology Karolinska Institutet Solna Sweden; ^33^ Function Perioperative Medicine and Intensive Care Karolinska University Hospital Stockholm Sweden; ^34^ Department of Clinical Science, Intervention and Technology Karolinska Institute Stockholm Sweden; ^35^ Department of Anaesthesia and Intensive Care Central Hospital Karlstad Sweden; ^36^ North Estonia Medical Centre Tallinn Estonia; ^37^ Department of Intensive Care Christchurch Hospital Christchurch New Zealand; ^38^ Middlemore Hospital Auckland New Zealand; ^39^ Department of Anesthesia and Intensive Care Medicine, Division of Emergencies and Critical Care Oslo University Hospital Oslo Norway; ^40^ Department of Anaesthesia and Intensive Care Medicine Lovisenberg Diaconal University College Oslo Norway; ^41^ Department of Intensive Care Medicine Stavanger University Hospital Stavanger Norway; ^42^ Department of Anaesthesia and Intensive Care Medicine Centre Hospitalier de Luxembourg Rollingergrund Luxembourg; ^43^ Department of Life Sciences and Medicine, Faculty of Science, Technology and Medicine University of Luxembourg Luxembourg City Esch‐sur‐Alzette Luxembourg; ^44^ University Heart Center Lübeck, University Hospital Schleswig‐Holstein Lübeck Germany; ^45^ Department of Neurology and Stroke University Hospital Tuebingen Tübingen Germany; ^46^ Department of Neurology and Stroke Hertie Institute of Clinical Brain Research Tübingen Germany; ^47^ Department of Neurology Charité – Universitätsmedizin Berlin Berlin Germany; ^48^ Department of Nephrology and Medical Intensive Care Charité – Universitätsmedizin Berlin Berlin Germany; ^49^ Department of Intensive Care Medicine Ghent University Hospital Ghent Belgium; ^50^ Department of Cardiology Ziekenhuis Oost‐Limburg Genk Belgium; ^51^ Adult Critical Care University Hospital of Wales Cardiff UK; ^52^ St George's Hospital London UK; ^53^ Critical Care and Perioperative Medicine Group Queen Mary University of London London UK; ^54^ Department of Perioperative Medicine, Bart's Heart Centre St. Bartholomew's Hospital London UK; ^55^ Wellcome‐Wolfson Institute for Experimental Medicine Queen's University Belfast Belfast UK; ^56^ Regional Intensive Care Unit Royal Victoria Hospital Belfast UK; ^57^ Department of Critical Care Medicine King's College Hospital London UK; ^58^ Guy's & St Thomas' NHS Trust London UK; ^59^ Intensive Care Unit University Hospitals Bristol and Weston Bristol; ^60^ CEDAR (Centre for Healthcare Evaluation Device Assessment and Research) Cardiff and Vale University Health Board Cardiff UK; ^61^ Essex Cardiothoracic Centre, MSE NHSFT Essex UK; ^62^ Anglia Ruskin School of Medicine & MTRC Essex UK; ^63^ Department of Critical Care Portsmouth University Hospitals Trust Portsmouth UK; ^64^ University College Dublin Clinical Research Centre at St Vincent's University Hospital, University College Dublin Dublin Ireland; ^65^ The Alfred Hospital Melbourne Australia; ^66^ Institute of Intensive Care Medicine University Hospital Zurich Zurich Switzerland; ^67^ Department of Intensive Care Medicine Inselspital University Hospital Bern Bern Switzerland; ^68^ Klinik für Intensivmedizin, Kantonsspital St. Gallen St. Gallen Switzerland; ^69^ Istituto Cardiocentro Ticino Lugano Lugano Switzerland; ^70^ Division of Intensive Care and Emergency Medicine, Department of Internal Medicine Medical University Insbruck Innsbruck Austria; ^71^ Dipartimento di Scienze Chirurgiche Diagnostiche Integrate IRCCS Policlinico San Martino, University of Genova Genova Italy; ^72^ Dipartimento di Scienze Chirurgiche Diagnostiche Integrate University of Genova Genova Italy; ^73^ Anaesthesia and Intensive Care Pordenone Hospital, Azienda Sanitaria Friuli Occidentale Pordenone Italy; ^74^ Faculty of Medicine Charles University in Prague Prague Czech Republic; ^75^ Department of Internal Medicine, Cardiovascular Medicine General University Hospital Prague Czech Republic; ^76^ Institute for Heart Diseases Wroclaw Medical University Wrocław Poland; ^77^ Department of Internal Medicine, Cardiology and Angiology General University Hospital in Prague Prague Czech Republic; ^78^ Department of Medicine, Department of Cardiovascular Medicine, First Faculty of Medicine Charles University in Prague and General University Hospital in Prague Prague Czech Republic; ^79^ Department of Intensive Care King Saud bin Abdulaziz University for Health Sciences and King Abdullah International Medical Research Center Riyadh Saudi Arabia; ^80^ Department of Anesthesia, Critical Care and Pain Medicine Jaber Alahmad Alsabah Hospital Zahra Kuwait; ^81^ Tan Tock Seng Hospital Singapore; ^82^ Yong Loo Lin School of Medicine National University of Singapore Singapore; ^83^ Lee Kong Chian School of Medicine Nanyang Technological University Singapore; ^84^ Intensive Care Unit Liverpool Hospital, South Western Sydney Local Health District Sydney New South Wales Australia; ^85^ South Western Clinical School University of New South Wales Sydney New South Wales Australia; ^86^ Department of Clinical Sciences The Ingham Institute for Applied Medical Research Sydney New South Wales Australia; ^87^ Intensive Care Unit, Liverpool Hospital South Western Sydney Local Health District Sydney New South Wales Australia; ^88^ Caboolture and Royal Brisbane and Women's Hospitals, Metro North Hospital and Health Service Brisbane Queensland Australia; ^89^ School of Clinical Medicine Queensland University of Technology Brisbane Queensland Australia; ^90^ Critical Care Division, The George Institute for Global Health University of New South Wales Sydney New South Wales Australia; ^91^ Medical School University of Queensland, Royal Brisbane and Women's Hospital Brisbane Australia; ^92^ Malcolm Fisher Department of Intensive Care Medicine Royal North Shore Hospital Sydney Australia; ^93^ Northern Clinical School, Sydney Medical School University of Sydney Sydney Australia; ^94^ Intensive Care Unit Tampere University Hospital Tampere Finland; ^95^ HUS Jorvi Hospital ICU Esbo Finland; ^96^ Research Unit of Translational Medicine, Research Group of Anaesthesiology, Medical Research Center Oulu Oulu University Hospital and University of Oulu Oulu Finland; ^97^ OYS Heart, Oulu University Hospital MRC Oulu and University of Oulu Oulu Finland; ^98^ Department of Intensive Care Kuopio University Hospitals Kuopio Finland; ^99^ Anesthesia and Intensive Care, Department of Clinical Sciences Lund Lund University Lund Sweden; ^100^ Intensive and Perioperative Care Skåne University Hospital Malmö Sweden; ^101^ Copenhagen Trial Unit, Centre for Clinical Intervention Research Copenhagen University Hospital – Rigshospitalet Copenhagen Denmark

**Keywords:** cardiac arrest, feedback‐controlled device, fever, randomized controlled trial, temperature management

## Abstract

**Background:**

Fever is associated with brain injury after cardiac arrest. It is unknown whether fever management with a feedback‐controlled device impacts patient‐centered outcomes in cardiac arrest patients. This trial aims to investigate fever management with or without a temperature control device after out‐of‐hospital cardiac arrest.

**Methods:**

The TEMP‐CARE trial is part of the 2 × 2 × 2 factorial *Sedation, TEmperature and Pressure after Cardiac Arrest and REsuscitation (STEPCARE)* trial, a randomized, international, multicenter, parallel‐group, investigator‐initiated, superiority trial that will evaluate sedation strategies, temperature management, and blood pressure targets simultaneously in nontraumatic/nonhemorrhagic out‐of‐hospital cardiac arrest patients following hospital admission. For the temperature management component of the trial described in this protocol, patients will be randomly allocated to fever management with or without a feedback‐controlled temperature control device. For those managed with a device, if temperature ≥37.8°C occurs within 72 h post‐randomization the device will be started targeting a temperature of ≤37.5°C. Standard fever treatment, as recommended by local guidelines, including pharmacological agents, will be provided to participants in both groups. The two other components of the STEPCARE trial evaluate sedation and blood pressure strategies. Apart from the STEPCARE trial interventions, all other aspects of general intensive care will be according to the local practices of the participating site. A physician blinded to the intervention will determine the neurological prognosis following European Resuscitation Council and European Society of Intensive Care Medicine guidelines. The primary outcome is all‐cause mortality at six months post‐randomization. To detect a 5.6% absolute risk reduction (90% power, alpha .05), 3500 participants will be enrolled. Secondary outcomes include poor functional outcome at six months, intensive care‐related serious adverse events, and overall health status at six months.

**Conclusion:**

The TEMP‐CARE trial will investigate if post‐cardiac arrest management of fever with or without a temperature control device affects patient‐important outcomes after cardiac arrest.

## 1 BACKGROUND AND SIGNIFICANCE

An estimated 3.8 million people will have an out‐of‐hospital cardiac arrest each year.^1^ Despite advances in medical care (medications external pacing and defibrillation etc.), only a minority of patients survive with a good functional outcome.^2^ Effective treatments in post‐cardiac arrest care are lacking, making even small improvements in outcomes clinically meaningful. One evolving strategy is temperature control, which gained attention after two trials in 2002 showed the benefit of hypothermia (32–34°C for 12–24 h) in improving survival and neurological outcomes for patients with shockable rhythms.^3^, ^4^

In 2013, the *Targeted Temperature Management at* 33 versus 36°C *after Cardiac Arrest trial* (the TTM‐trial) compared 33 and 36°C temperature management for 36 h in 950 OHCA survivors but found no significant differences in patient outcomes between the two strategies.^5^ The TTM2 trial in 2021, with 1900 participants, did not find an advantage with hypothermia and noted increased arrhythmias and other complications.^6^ Meta‐analyses of these trials support these findings.^7^ The European Resuscitation Council (ERC) and European Society of Intensive Care Medicine (ESICM) find insufficient evidence to recommend for or against specific temperature targets, suggesting instead to prevent fever (>37.7°C) for at least 72 h.^8^, ^9^ In contrast, the American Heart Association's 2023 guidelines recommend maintaining temperatures between 32 and 36°C for 24 h post‐cardiac arrest.^10^ After the publication of these guidelines, several recent systematic reviews have found a lack of evidence to support hypothermia in the manner it is currently performed in the ICU.^11^, ^12^, ^13^, ^14^, ^15^

Fever, common after cardiac arrest due to neurological damage, inflammation, or infection, is associated with brain injury and poor outcomes, as suggested by animal and observational studies.^16^, ^17^, ^18^ However, temperature management with a feedback‐controlled device is resource‐intensive and carries risks of morbidity such as increased arrhythmias.^19^, ^20^, ^21^ It also impacts blood clotting, increasing risks of hemorrhage and thromboembolism, especially in patients on anticoagulants.^22^, ^23^, ^24^ Supporting temperature management, a meta‐analysis showed TTM to be beneficial under most experimental conditions in animal models of cardiac arrest or global brain ischemia.^25^ However, a Finnish study found no association between fever and outcome in patients not treated with TTM after OHCA.^26^ Further, a Danish study showed no significant outcome differences between patients who received device‐based temperature control for 36 h and those who received it for 72 h post‐cardiac arrest.^27^ Another study, assessing fever treatment in patients with acute vascular brain injury, showed no benefit of the surface cooling device on patient‐centered outcomes compared with standard treatment.^28^ Furthermore, a systematic review found no strong evidence supporting fever therapy to reduce all‐cause mortality.^29^ In large parts of Scandinavia, the current clinical practice includes post‐OHCA fever prevention, as recommended by local guidelines.^30^, ^31^ The discordance between guidelines reflects ambiguity in interpreting available evidence, highlighting the need for more research.

Here, we describe the Temperature after Cardiac Arrest and Resuscitation (TEMP‐CARE) trial, which is part of the factorial STEPCARE randomized clinical trial. The two other interventions of the STEPCARE trial (Sedation and Blood pressure) are described separately.

## 2 METHODS

### 2.1 Trial design

The TEMP‐CARE trial is registered at clinicaltrials.gov (NCT05564754, 2022‐10‐03) as part of the 2 × 2 × 2 factorial STEPCARE trial. The STEPCARE trial protocol was designed following the SPIRIT guidelines,^32^ and the trial will be reported according to the CONSORT guidelines.^33^, ^34^ The full STEPCARE trial protocol is available at www.stepcare.org. The STEPCARE trial is a randomized international, multicenter, parallel‐group, investigator‐initiated, superiority trial with three simultaneous intervention arms, considered three separate trials. In TEMP‐CARE, participants will be randomized to cardiac arrest management with or without a temperature control device. In the two other interventions, participants will be randomized to continuous deep sedation or minimal sedation and a mean arterial pressure target of >85 mmHg or >65 mmHg. All participants will be included in all three parts of the STEPCARE trial, and selective participation in TEMP‐CARE is not allowed. Apart from the interventions of the STEPCARE trial, intensive care management will be according to the local practices of each participating hospital.

### 2.2 Inclusion criteria

We will include adults (≥18 years) who experience an OHCA with a sustained return of spontaneous circulation (ROSC; 20 min or more of spontaneous circulation without the need for chest compressions) and who are unconscious, defined as not being able to obey verbal commands (Full Outline of UnResponsiveness [FOUR] score motor response <4),^35^ or who are intubated and sedated because of agitation after sustained ROSC. Participants must not have treatment limitations for intensive care (e.g., a “do not attempt resuscitation” order or a decision not to escalate care) to be included in the trial. Screening will be performed as soon as possible but no later than 240 min after sustained ROSC.

### 2.3 Exclusion criteria

Exclusion criteria will be trauma or hemorrhage (including gastrointestinal bleeding) as the presumed cause of the arrest, pregnancy, suspected or confirmed intracranial hemorrhage, and previous randomization to the STEPCARE trial. Additionally, patients treated with extracorporeal membrane oxygenation (ECMO) prior to randomization will be excluded.

### 2.4 Screening and randomization

Screening will be performed in the emergency room, angiography suite, or the intensive care unit (ICU). Clinical investigators at each participating site will be responsible for screening all patients resuscitated from an OHCA. A screening log will be compiled, including all cardiac arrest patients with sustained ROSC admitted to the ICU, to document whether they are eligible for inclusion. Informed consent will be obtained according to national ethical approvals. The reason for the exclusion of screened patients will be documented and reported. Randomization will be performed via a web‐based application to allow immediate allocation to treatment groups and ensure allocation concealment and adequate allocation sequence generation. Randomization will be performed with blinded permuted blocks of varying size, stratified for trial site.

### 2.5 Intervention

In the TEMP‐CARE trial, participants will be randomly assigned to fever management with or without a feedback‐controlled surface or intravascular temperature control device. Core body temperature will be continuously measured, preferentially via a bladder catheter, but an alternate core temperature site, such as the esophagus or blood (central circulation) will be allowed.

For participants allocated to fever management with a device, temperature management devices will be started to achieve a core body temperature of ≤37.5°C if the temperature reaches the threshold of ≥37.8°C before 72 h after randomization. If needed, pharmacological agents and other standard ICU fever treatments will also be administered alongside device‐based management. For participants allocated to fever management without a device, fever will be managed as per standard fever treatment in the ICU, including measures such as exposure control and pharmacological agents. In cases where participants in the fever management without a device group experience a very high, treatment‐refractory fever deemed to compromise the patient, the treating physician can decide to apply a device to lower the temperature. This will be considered as bailout device management. All usage of device‐based cooling in fever management without a device group will be documented and reported.

The intervention will continue for 72 h after randomization or until extubation, whichever occurs first. After this intervention period and after ICU discharge, temperature management will be at the discretion of the treating physician. The allocated fever management will be re‐established if a participant is reintubated during the 72‐h intervention period. If the participant is assessed for brain death according to national criteria, fever management will be determined by the clinician.

### 2.6 General intensive care

General intensive care, including management of respiration, metabolic disturbances, ulcer prophylaxis, deep venous thrombosis prophylaxis, and other aspects of intensive care, should be delivered similarly in all allocation groups and according to local protocols at the discretion of the treating physicians. Cardiac interventions will also be guided by local protocols. However, participating centers will need to have access to around‐the‐clock invasive management, either on‐site or at a nearby hospital, which is also part of the trial. Cardiac catheterization (coronary angiography) should not be delayed by the trial interventions. Apart from the interventions, adhering to international and national guidelines for post‐resuscitation care is recommended.

### 2.7 Blinding

The clinical team responsible for the immediate care of the participant will not be blinded to the study interventions due to inherent difficulty in blinding the interventions (sedation, temperature, and arterial pressure). Measures will be taken to ensure that allocation information will be disseminated only within the immediate group of healthcare workers responsible for patient care. A physician blinded to the intervention will make a first prognostic evaluation of the participant 72 h after randomization and make a statement on neurological prognosis (for details, see below).

Participants, their legal representatives, and family will only be informed that the patient has been part of the trial, but not the allocation group. The outcome assessors, prognosticators, statisticians, the data safety monitoring committee (DSMC), members of the steering committee, and authors of the manuscript will be blinded to treatment allocation. The intervention groups will be coded as “X” and “Y”. Two abstracts will be prepared, one assuming X is the experimental group and Y is the control group, and one assuming the opposite. The author group must approve conclusions before the randomization code is broken.

### 2.8 Prognostication and withdrawal of life‐sustaining therapies

The trial will employ a conservative and strict protocol for neurological prognostication according to the European Resuscitation Council (ERC) and the European Society of Intensive Care Medicine (ESICM) recommendations (see Supporting Information).^8^, ^36^ Prognostication will be performed on all participants who are not awake and obeying verbal commands and who are still in the ICU at 72 h after randomization. Prognostication will be made by a physician experienced in neuro‐prognostication after cardiac arrest and blinded to treatment allocations. The external physician, blinded to the intervention, will not make recommendations about withdrawal of life‐sustaining measures.

Presumed poor functional outcome will not justify the withdrawal of life‐sustaining therapies (WLST) prior to prognostication. Life‐sustaining therapies may only be withdrawn before protocolized prognostication in the following situations: information on a pre‐existing advanced care directive or an advanced medical comorbidity (e.g., generalized malignant disease) that prohibits continuation of care becomes available after inclusion in the trial or if continuation of care is considered unethical due to irreversible multiorgan failure. Brain death, established according to local legislation, will be defined as death and not WLST. See Supporting Information for a detailed description of neurological prognostication and WLST.

### 2.9 Follow‐up

Long‐term outcomes will be collected during a telephone follow‐up at 30 days and six months after randomization. The blinded outcome assessor, blinded to the intervention, may be an occupational therapist, physician, research nurse, psychologist, or another health care professional. The central follow‐up coordinating team will provide outcome assessors with detailed guidelines and study‐specific training. More detailed outcomes will be collected in an extended follow‐up sub‐study at selected sites including, for example, cognitive function, societal participation, and family impact. This sub‐study is described elsewhere.

### 2.10 Outcome measures

The primary and secondary outcomes will be assessed six months after randomization. The primary outcome will be all‐cause mortality. Secondary outcomes will be the proportion of participants with a poor functional outcome defined primarily as a score of 4–6 (moderately severe disability, severe disability, or dead) reported by the structured modified Rankin Scale (mRS, range 0–6 with higher scores indicating a worse outcome). If an mRS score cannot be assigned, patients will be categorized based on whether they are dependent on others for basic activities of daily life (need of assistance with, for example, moving indoors, eating, dressing, taking care of personal hygiene), similar to an mRS score of 4–6 but without the detailed information that is needed to separate outcomes between categories. Other secondary outcomes will include the proportion of patients who died or had a pre‐defined serious adverse event in the ICU, and patient‐reported overall health by using the EQ visual analog scale (EQ VAS), a part of the EQ‐5D.

Exploratory outcomes will be ventilator‐free days within the first 30 days, hospital‐free days within the first 30 days, mRS (ordinal score), time‐to‐event and win ratio (dead vs. alive), all steps on the mRS scale, safety event, and detailed information from the EQ‐5D‐5L.

### 2.11 Adverse events

It is recognized that the intensive care patient population will experience several common aberrations in laboratory values, signs, and symptoms due to the severity of the underlying disease and the impact of standard therapies. Intensive care patients will frequently develop life‐threatening organ failure(s) unrelated to study interventions. Therefore, consistent with established practice in academic ICU trials,^37^ events that are part of the natural history of the primary disease process or expected complications of critical illness will not be reported as adverse events in this study. All adverse events potentially causally related to the study intervention or that are otherwise of concern in the investigator's judgment will be reported and reviewed by the management committee and the DSMC. Several specified serious adverse events (as described below) are captured in the trial case report form and will not be reported as SAEs.

Only predefined serious adverse events (Table 1) and any unexpected serious adverse event will be reported by the investigator to avoid over‐reporting and to maximize the probability of finding “true” and important differences. Pre‐defined serious adverse events were selected based on commonly occurring adverse events observed in previous trials investigating temperature control after cardiac arrest.^5^, ^6^

**TABLE 1:** Definition of specific serious adverse events.

Serious adverse event	Definition
Sepsis or septic shock	Sepsis‐3 criteria^38^
Arrhythmia	Arrhythmia requiring defibrillation or chest compressions
Moderate or severe bleeding	GUSTO criteria^39^

Abbreviation: GUSTO, global utilization of streptokinase and tissue plasminogen activator for occluded coronary arteries.

### 2.12 Rationale for chosen outcomes

All‐cause mortality was chosen as the primary outcome to ensure an unbiased assessment and to avoid competing risks. We will use the mRS to evaluate functional outcome. The mRS scale is increasingly used in cardiac arrest research and is currently recommended by the Core Outcome Set for Cardiac Arrest (COSCA) and the International Liaison Committee on Resuscitation (ILCOR) consensus statement for measuring functional outcome after cardiac arrest.^40^ The primary analysis will be a binary analysis, with the mRS dichotomized as 0–3 (none to moderate disability) versus 4–6 (moderately severe disability to death), as this dichotomization separates patients that are nondependent from patients that are dependent on others in basic activities of daily living. This dichotomization has also been used historically in cardiac arrest trials.^41^ If mRS is not available, binary dependency rating will be used.

The EQ‐VAS included as a part of EQ‐5D‐5L will be used to measure a patient‐reported outcome of overall health status. This instrument was chosen since it is easy to use, has shown evidence to be a valid measure in many situations, and could be used as a proxy report if necessary.^42^ We will measure possible harmful effects of the intervention by predefined serious adverse events that are most common and plausibly related to the intervention. A more detailed description of the rationale for chosen outcomes is available in the Supporting Information.

### 2.13 Factorial design

Factorial trials have the inherent risk of potential interactions between interventions on both physiological and patient‐centered outcomes.^43^ This trial is conducted assuming no meaningful interaction between the interventions on specified patient‐centered outcomes. The TEMP‐CARE trial intervention has potential physiological interactions with the sedation and blood pressure target interventions of the STEPCARE trial. Targeting deep sedation may induce further vasodilation and subsequently lower body temperature after cardiac arrest, potentially impacting the attainment of the designated temperature target. Elevated doses of vasopressors could lead to reduced heat dissipation through vasoconstriction, thereby increasing the necessity for active fever management. Nevertheless, there is currently no evidence to suggest that these interactions could influence the evaluated outcomes. If heightened doses or levels of sedation, inotropic/vasopressor support, or external cooling are necessitated due to between‐group interactions, distinct adverse effects from these interactions could theoretically occur. The DSMC will monitor the trial during its conduct to identify possible interaction effects on outcomes, focusing on patient safety. For more specific situations of intervention interaction see Supporting Information.

### 2.14 Co‐enrolment in other trials

Trial participants may be included in any observational study that does not affect protocol adherence in the STEPCARE trial. We will assess co‐enrollment suggestions based on the Spice‐8 co‐enrolment guidelines.^44^ Unless there are clear conflicts between trial interventions, co‐enrollment in other randomized trials will be possible. The STEPCARE management committee will assess co‐enrollment on a case‐by‐case basis.

### 2.15 Data collection and management

Individual patient data regarding background characteristics, clinical features, and laboratory results will be obtained from medical records, ambulance service, and relatives. We will collect detailed data, including neurological status, body temperature, blood pressure values, and doses of vasoactive and sedative medications. Detailed information on collected data is described in the Supporting Information. Data will be entered into a web‐based electronic case report file (eCRF) by site personnel. The software for eCRF is provided by Spiral, New Zealand, but the storage server for the trial database is handled by the trial's coordinating team.

### 2.16 Sample size and power estimations

The sample size estimation is based on a 60% mortality in the control arm and a 54.4% mortality in the intervention arm at six months, referring to the results of the TTM‐trial,^5^ TTM2‐trial,^6^ and the international cardiac arrest registry (INTCAR).^45^ To demonstrate a relative risk of 0.91 with 90% power at a significance level of 0.05, using two‐sided tests, 1639 participants are required in each group, a total of 3278 participants. In the TTM2 trial,^6^ loss to follow‐up was approximately 2% and we expect a similar loss to follow‐up in the STEPCARE trial. Therefore, the sample size was increased by 6.8% to 3500 participants. 1.8% of the increment is considered to account for loss to follow‐up and, as a pragmatic choice, 5% is considered to account for possible interactions between interventions on patient‐centered outcomes. The sample size calculation corresponds to a relative risk reduction of 9.3%, and an absolute risk reduction of 5.6%, which we submit is a clinically relevant and realistic treatment effect. For the secondary outcomes, we estimated a power of 91% to detect a relative risk reduction of 0.9 for poor outcomes (mRS 4–6), a power of >90% to detect a difference of five points on the EQ‐5D‐5L VAS‐scale, and a power of 91% to detect a relative risk reduction or increase of 10% for the predefined serious adverse events (Table 1).

### 2.17 Statistical analyses

All analyses will be conducted according to the intention‐to‐treat principle and adjusted for ‘site’ and the allocated intervention in the two other trials of the factorial STEPCARE trial. Dichotomous outcomes will be presented as proportions of participants with the event and relative risks with 95% confidence intervals. Continuous outcomes will be presented as means and standard deviations for each group, with 95% confidence intervals for the means of the groups and the mean differences between the groups. Count data will be presented as means, mean differences, and 95% confidence intervals or medians and interquartile ranges depending on the observed distribution. Dichotomous outcomes will be analyzed using mixed‐effects generalized linear model, continuous outcomes using mixed effects linear regression model, and count data using the Wilcoxon test. Mock tables, curves, and graphs presenting characteristics of the participants, results, and separation of blood pressure and vasopressor dose between the groups are provided in the Supporting Information. A detailed statistical analysis plan will be published separately.

### 2.18 Subgroup analysis

The following subgroup analyses will be performed:

- Age (<median or ≥median)
- Sex (male/female)
- Witnessed arrest
- Bystander cardiopulmonary resuscitation (yes/no)
- Initial rhythm (shockable vs. nonshockable)
- Time to ROSC (<median or ≥median)
- Circulatory status on admission (presence or absence of circulatory shock diagnosed by the treating physician)
- Baseline risk of poor functional outcome (Miracle2‐score: low risk [0–2], medium risk [3–5], and high risk [6–10])^46^
- Presumed cause of cardiac arrest at randomization (cardiac vs. others), and
- Diagnosed with chronic hypertension (yes/no).

### 2.19 Ethics and informed consent

Ethics application will seek approval for a delayed written consent process since the intervention must be regarded as an emergency procedure and must be started as soon as the participants are admitted to hospitals. Participants regaining consciousness will be asked for written consent as soon as they are able to make an informed decision. It is of importance that ethical approval contains a request to use data also for deceased participants, to avoid survival bias. The consent process will vary from site to site and align with ethics approval, national law, and the Declaration of Helsinki.^47^

### 2.20 Data safety monitoring and interim analysis

The Charter for the DSMC of the STEPCARE trial describes the role and function of the DSMC. The primary focus of the DSMC is monitoring the safety and efficacy of the interventions and overall conduct of the trial to guard the interests of the trial participants. The first interim analysis was conducted after the enrollment of 500 participants, with the recommendation from the DSMC to *continue the trial as planned*. The schedule of further interim analyses will be decided by the DSMC, but a minimum of three interim analyses will be conducted. The DSMC will arrange for an independent statistician to conduct a blinded interim analysis. The DSMC can request the unblinding of data if required. The survival and safety parameters are provided for the DSMC for the conduction of the interim analyses. Lan‐DeMets group sequential monitoring boundaries will be used as the statistical limit to guide recommendations regarding the early termination of the trial.^48^ Interventions of the STEPCARE trial will not be stopped for futility. The DSMC may recommend stopping or pausing the TEMP‐CARE trial, or the entire STEPCARE trial if:

- Group difference in the primary outcome measure is found in the interim analysis according to pre‐defined stopping rules;
- Group difference in serious adverse events is found in the interim analysis;
- Evidence of interaction influencing outcomes; or
- Results from other studies show benefit or harm with one of the allocation arms.

It is the steering group's decision whether the trial should be stopped.

### 2.21 Patient group involvement

We followed the COSCA guidelines, developed in collaboration with ILCOR, which involved patient representatives to facilitate the selection of patient‐centered outcomes.^41^ Patient organizations in Sweden and Australia were involved in the design phase of the TEMP‐CARE trial.

### 2.22 Trial status and timeline

Randomization began in August 2023 and trial sites have been added gradually. We estimate that the last six‐month follow‐up will be performed during 2026–2027. Results from each intervention and sub‐study will be reported separately. The initial publication for the TEMP‐CARE trial will include results of primary and secondary outcomes.

## 3 DISCUSSION

The TEMP‐CARE trial, embedded within the larger STEPCARE platform, aims to investigate the impact of using a temperature control device versus standard fever management in unconscious adults resuscitated from OHCA. We will assess the impact on survival, functional outcome, and overall health status. Further, we will investigate the incidence of treatment‐related serious adverse events.

Despite advancements in resuscitation and intensive care, only a minority of patients have a good outcome.^49^ The STEPCARE trial seeks to investigate multiple treatments of high interest. The absence of effective treatments for unconscious survivors of OHCA underscores the critical need for research in this area. The TEMP‐CARE trial addresses this gap by evaluating the efficacy of different fever management strategies in a novel approach.

A trial published in 2023 examined the efficacy of device‐based fever prevention for 36 h versus no device‐based fever prevention, after all patients had received 36 h of temperature control to 36°C. Results revealed no significant difference in mortality or severe disability between the groups (32.3% vs. 33.6%), with similar cognitive scores observed.^27^ These findings suggest that prolonging fever prevention beyond 36 h does not yield discernible benefits post‐cardiac arrest; however, the population had an overall mortality indicating less severity of injury, and the results may not be fully generalizable.

Relevant to our trial, the INTREPID trial demonstrated that preventive normothermia using an automated surface temperature management device effectively reduced fever burden in patients with acute vascular brain injury but did not result in improved functional outcomes.^28^ While controlling fever appears feasible, the lack of functional benefit observed in the INTREPID trial raises questions about the clinical significance of fever control in improving outcomes. This underscores the importance of further exploring whether targeted temperature management in our specific patient population can yield meaningful improvements in survival and neurological recovery.

The rationale for investigating fever management stems from its potential neuroprotective effects and conflicting evidence from previous trials. By including fever management as a key intervention, the TEMP‐CARE trial addresses an important aspect of post‐cardiac arrest care that has not been extensively studied in large clinical trials before. The broad inclusion criteria ensure that a diverse population of OHCA patients is represented, allowing for the generalizability of the findings.

## 4 STRENGTHS AND LIMITATIONS

One of the strengths of the TEMP‐CARE trial is its large sample size and predefined methodology, which minimize the risk of bias and allow for the detection of small but clinically meaningful effects. Furthermore, patient‐centered outcomes, such as functional status and overall health status, are prioritized, ensuring that the trial outcomes are relevant to patients and clinicians alike. Broad inclusion criteria further enhance the generalizability and external validity of the results.

Interactions between the blood pressure, sedation, and temperature strategies, with an effect on patient‐centered outcomes, are a possibility and must be considered a limitation. This risk is implicit in all factorial trials. We have designed the study assuming that there is no interaction on the primary, secondary, and exploratory endpoints with the three strategies. Following calculation of the sample size, we have allowed for a small increment of the sample size (6.8%) to allow for loss to follow‐up and a small interaction effect.

Having open‐label temperature targets is another limitation in the TEMP‐CARE trial, similar to previous hypothermia trials. However, measures have been taken to minimize its possible impact on the results and strategies to ensure that we are kept blinded by blinding the steering group, authors of the manuscript, outcome assessors, prognosticators, statisticians, and the data safety monitoring committee.

Allowing for standard fever management in the no device (control) arm will lessen the separation between the two intervention groups in terms of temperature, but there may still be a clear separation in terms of the use of the device. Since the natural trajectory of body temperature and fever after cardiac arrest is poorly studied, there may after the STEPCARE trial be residual knowledge gaps regarding whether fever is detrimental or just an epiphenomenon related to the severity of brain injury.

## 5 CONCLUSION

In conclusion, the TEMP‐CARE trial will advance our understanding of post‐cardiac arrest care fever management strategies. With a large sample size and a broad patient population, the results of this trial will inform clinical practice guidelines and have the potential to improve outcomes for unconscious survivors of OHCA.

## AUTHOR CONTRIBUTIONS

J. Holgersson drafted the manuscript. All other authors contributed to the study design and critically revised the manuscript. All authors approved the final version.

## FUNDING INFORMATION

The trial is funded by The Swedish Research Council, ALF‐project funding within the Swedish Health Care, The Academy of Finland, The Sigrid Jusélius Foundation (Finland), Hospital district of Helsinki and Uusimaa (Finland), Medicinska Understödsföreningen Liv och Hälsa (Sweden), Medical Research Future Fund (Australia), Health Research Council of New Zealand, and the Clinical Research Programme Directorate of Health Ministry of Health and Social Security (Luxembourg).

## CONFLICT OF INTEREST STATEMENT

Skrifvars MB received a speakers fee from BARD Medical (Ireland) 2022 and is a member of the editorial board of Acta Anaesthesiologica Scandinavica. Leithner C has received research support from the Laerdal Foundation. Nee J received honorarium and travel costs for presentations from BD BARD and Xenios AG. Joannidis M has received consulting fees from Baxter Healthcare Corp, AM‐Phjarma, Biomerieux, Sphingotec and AOP pharma, and honoraria for lectures from Baxter Healthcare Corp, AOP Health and Biomerieux, and grants from Baxter Healthcare and Fresenius. Pellis T has received lecture fees from BD. Reade M is an investigator in several National Health and Medical Research Council—funded trials, including trials of sedative agents and other treatments for brain injury.

## Supporting information


**Data S1:** Supporting information.

## Data Availability

Data sharing is not applicable to this article as no new data were created or analyzed in this study.
