# Supplementary material for: Fever management with or without a temperature control device after out‐of‐hospital cardiac arrest and resuscitation (TEMP‐CARE): A study protocol for a randomized clinical trial
Source: Acta Anaesthesiol Scand. 2025 Apr 13;69(5):e70034. doi: 10.1111/aas.70034 (PMC11994252; doi:10.1111/aas.70034)
Supplement: Supplementary file 1 — Data S1: Supporting information. [file AAS-69-0-s001.docx]

**Fever management with or without a temperature control device after out of hospital cardiac arrest and resuscitation (TEMP-CARE): A study protocol for a randomized clinical trial**

**Supplementary information**

Table of contents

[Prognostication and withdrawal of life sustaining therapies 3](#_Toc184147089)

[Rationale for chosen outcomes 3](#_Toc184147090)

[Data collection 4](#_Toc184147091)

[Baseline data 4](#_Toc184147092)

[Pre-hospital data 5](#_Toc184147093)

[Background data 5](#_Toc184147094)

[Data on hospital admission 6](#_Toc184147095)

[Data in the intensive care unit 6](#_Toc184147096)

[At ICU discharge 7](#_Toc184147097)

[At hospital discharge 9](#_Toc184147098)

[30 days after randomization 10](#_Toc184147099)

[6 months after randomization 10](#_Toc184147100)

[Biomarker substudy 10](#_Toc184147101)

[Table 1. Reasons for deviation from the allocated temperature management strategy 10](#_Toc184147102)

[Table 2. Criteria for a likely poor neurological outcome in the STEPCARE trial (according to ERC/ESICM recommendations)^5^ 11](#_Toc184147103)

[Table 3. Characteristics of the participants 12](#_Toc184147104)

[Table 4. Intervention-specific information 13](#_Toc184147105)

[Table 5. Primary and secondary outcomes, and predefined serious adverse events. 13](#_Toc184147106)

[Figure 1. Mock-figure of geometric means of temperature over time with 95% confidence interval (CI) in the fever management without a device and fever management with a device groups. 14](#_Toc184147107)

[Figure 2. Mock-figure of mean temperature ± 2 SD in the fever management without a device and fever management with a device groups. Values shown are medians and error bars indicate 2 standard deviations. 15](#_Toc184147108)

[Figure 3. Mock-figure of Kaplan-Meier estimates of the probability of survival until 180 days after randomization in the fever management without a device and fever management with a device groups. 15](#_Toc184147109)

# Prognostication and withdrawal of life sustaining therapies

The STEPCARE trial will employ a conservative and strict protocol for neurological prognostication according to the ERC and European Society of Intensive Care Medicine (ESICM) recommendations.^1,2^ Prognostication will be performed on all participants who are not awake and obeying verbal commands, and who are still in the ICU at 72 hours after randomization. The clinical examination used for prognostication may not be performed earlier than 72 hours after cardiac arrest but may be delayed due to practical reasons (such as weekends or national holidays). The physician performing the prognostication will be a neurologist, intensivist or other specialist experienced in neuro-prognostication after cardiac arrest and who has not been involved in the care of the patient. The prognosticator will be blinded for treatment allocations, but not for relevant clinical data. Prognostication will be based on the results of clinical examinations, neurophysiology, biomarkers of brain injury and imaging.

The result of the prognostication will be categorized as “YES” or “NO”, based on the answer to the question *“Does this patient fulfil the ERC/ESICM criteria for a likely poor neurological outcome?”*. This assessment will be recorded and communicated to the treating clinician (for details, see below).

The results of neurological prognostication and the potential decision to withdraw from active intensive care are closely related but will be considered separate entities. When the neurological outcome is considered poor, a decision to withdraw from active intensive care is made by the treating physician, the patient’s relatives and legal surrogates as required by local legislation. In making this decision, the treating physician may use the information from the prognostication. Prognostication must be sufficiently delayed, ensuring that any lingering effects of sedative agents will not affect the assessment. The blinded external physician will not make specific recommendation about withdrawal of life sustaining measures.

Presumed poor neurological outcome will not justify the withdrawal of life-sustaining therapies prior to prognostication. Life-sustaining therapies may be withdrawn before protocolized prognostication if information about a pre-existing Advanced Care Directive or advanced medical comorbidity (e.g. generalized malignant disease) becomes available after inclusion in the trial or continuation of care is considered unethical due to irreversible multi-organ failure. Brain death, established according to local legislation, will be defined as death and not withdrawal of life-sustaining therapy.

# Rationale for chosen outcomes

All-cause mortality was chosen as the primary outcome to ensure an unbiased assessment and to avoid competing risks. Although the interventions are primarily thought to affect the development of brain injury, mortality is a global assessment of the interventions’ effect on all organ systems. The estimated 60% mortality of the target population yields a high power to detect differences in a reasonably sized trial.

We recognize the risk that clinically relevant effects on the development of brain injury may be missed using mortality as the only outcome, as neurological outcome for out-of-hospital cardiac arrest survivors ranges from a vegetative state to complete recovery.

To complement the primary outcome, we will use the mRS-scale to evaluate functional outcome. The modified Rankin scale (mRS) is increasingly used in cardiac arrest and is currently recommended in a consensus statement as part of the Utstein template by the International Liaison Committee on Resuscitation (ILCOR). To facilitate clinical interpretation of the trial results, and to provide an understandable effect size, the primary analysis will be performed as a binary analysis, with the mRS-scale dichotomized (0-3 vs. 4-6). The scale is also part of the Core Outcome Set for Cardiac Arrest (COSCA) trials, which was developed by an ILCOR consensus group including patient and partner representatives.^3^ We are aware that stroke trials commonly use favorable outcome defined as mRS 0-2. To allow for a meaningful comparison of our results to previous cardiac arrest trials, we will define favorable outcome as mRS 0-3, as this dichotomization is historically used in cardiac arrest trials.^3^ However, we will also enable an exploratory ordinal mRS scale analysis to reveal differences in functional outcome which are not apparent in a dichotomous analysis.

It is of utmost importance to assess both beneficial and harmful effects of any intervention. We have, therefore, predefined serious adverse events, including the most common and those most plausibly related to the interventions.

To include patient-reported outcome measures, HRQoL is recommended by guidelines for outcome reporting after cardiac arrest and is part of COSCA’s recommendations. The EQ-5D-5L reported as the EQ-VAS was chosen as the trial’s HRQoL instrument since it is easy to use, validated, performs sufficiently well when obtained by proxy and may be used to calculate quality-adjusted life-years.^4^

An intervention might be associated with an increased short-term mortality but have a protective effect over time. We will therefore include survival assessed as time-to-event in an exploratory analysis.

# Data collection

## Baseline data

- Pre-randomization characteristics:
  - Inclusion and exclusion criteria
  - Age
  - Sex
  - Time and date of Cardiac Arrest
  - Time and date of return of spontaneous circulation (ROSC)
- Miracle 2 SCORE:
  - Witnessed arrest / Unwitnessed arrest
  - Shockable initial rhythm (yes/no)
  - Pupillary reflex (yes/no)
  - Age
  - Changing rhythms (Any two of ventricular fibrillation (VF)/pulseless electric activity (PEA)/asystole) (yes/no)
  - First available pH (arterial)
  - Epinephrine given (yes/no)
- Presumed cause of arrest:
  - Cardiac – ST-elevation myocardial infarction / acute coronary occlusion
  - Cardiac – non-ST elevation myocardial infarction
  - Cardiac – Arrhythmia – not related to acute ischemia
  - Cardiac – Heart Failure
  - Cardiac – Other cardiac
  - Pulmonary Embolism
  - Hypoxia
  - Other medical causes (electrolyte disorders, sepsis etc.)
  - Asphyxia (strangulation, foreign body etc.)
  - Drowning
  - Drug Overdose
  - Trauma/Bleeding
  - Intracranial bleed
- Perceived prognosis by randomizing physician (good or poor neurological function)

## Pre-hospital data

- Scene of arrest (home, work, public place, nursing facility, ambulance, other)
- Witnessed arrest (yes/no)
- Bystander cardiopulmonary resuscitation (CPR) (yes/no)
- First monitored rhythm at arrival of emergency medical services (asystole, PEA, VF, non-perfusing ventricular tachycardia, ROSC after bystander defibrillation, unknown (shockable or unshockable)
- Time of emergency call
- Minutes until CPR started (no-flow time)

## Background data

- Height
- Weight
- Pre-arrest functional status (Independent / Not independent, in basic activities of daily life)
- Previous percutaneous coronary intervention (yes/no)
- Previous coronary artery bypass grafting (yes/no)
- Previous heart failure with pharmacological treatment (yes/no)
- Previous implantable cardioverter defibrillator (ICD) (yes/no)
- Previous hypertension with pharmacologic treatment (yes/no)
- Previous diabetes mellitus (yes/no)
- Previous stroke or transitory ischemic attack (yes/no)
- Previous COPD (Chronic obstructive pulmonary disease)
- Pre-arrest frailty using the Clinical Frailty Score (1-9)
- Previous Kidney Disease (CKD4, eGFR<30)

## Data on hospital admission

- Time of ICU admission (day, hour)
- First recorded tympanic temperature (bilateral, highest value)
- Full Outline of UnResponsiveness (FOUR) motor score
- Preserved pupillary reflexes
- Preserved corneal reflexes
- First lactate (arterial or venous)
- First creatinine
- Highest outpatient (not in hospital) creatinine measured during the previous 6 months before this cardiac arrest
- First troponin
- STEMI - New ST-segment elevation ≥1 mm in ≥2 contiguous ECG leads or posterior STEMI
- ECG rhythm (atrial fibrillation or flutter, sinus, other)
- Shock on admission, Systolic blood pressure <90mmHg for at least 30 minutes or the need for supportive measure to maintain a systolic blood pressure ≥90mmHg and end-organ hypoperfusion (cool extremities, or urine output of less than 30ml/hr, and a HR >60 beats per minute
- Severity of Shock (SCAI class) (Beginning, Classic, Deteriorating, Extremis)
- Echocardiography performed during first 24h (yes/no)
- Left Ventricular Ejection Fraction (Normal > 55%, mildly reduced 40-54%, moderately reduced 30-39%, severly reduced < 30%, EF not reduced)
- Depressed Right Ventricular function (yes/no)
- Other Pathology: severe aortic stenosis, severe mitral regurgitation, severe tricuspid regurgitation, regional wall motion abnormality, none.

## Data in the intensive care unit

*At 0, 2, 4, 6, 8, 10, 12, 14, 16, 18, 20, 22, 24, 28, 32, 36, 40, 48, 56, 72, 96, and 120 hours:*

- Core temperature (bladder, rectal, or esophageal probe)
- Systolic, diastolic, and mean arterial pressure
- Heart rate

*At 0, 4, 8, 12, 16,20,24,28,32,36,40,48,72, 96, and 120 hours:*

- Richmond Agitation-Sedation Sclae (RASS) score
- Propofol dose (mg/kg/h)
- Dexmedetomidine dose (mcg/kg/h)
- Noradrenaline dose (mcg/kg/min)
- Midazolam infusion (yes/no)
- Dobutamine infusion (yes/no)
- Adrenaline infusion (yes/no)
- Responds to commands (yes/no)

*At 0,12, 24, 36, 48, 72, 96, and 120 hours:*

- Mechanically ventilated (check if yes)
- Arterial oxygen tension
- Arterial carbon dioxide tension
- Lactate (arterial)
- pH
- Respiratory rate
- Peak end-expiratory pressure
- Tidal volume
- Pressure control, volume control, or pressure support/CPAP ventilation
- Plateau pressure (for volume control)
- Inspiratory pressure (for pressure control)
- Fraction of inspired oxygen
- Arterial oxygen saturation
- Highest Bed Side Shivering Assessment Scale score
- FOUR motor score
- Presence of status myoclonus
- Presence of corneal and pupillary reflexes
- Any tonic/clonic seizures during the previous 24h
- Any status myoclonus seizures during the previous 24h
- Highest ICU Mobility Scale previous 24h
- Delirium (assessed using Confusion Assessment Method for the ICU (CAM ICU) or Intensive Care Delirium Screening Checklist (ICDSC) in the previous 24h (yes/no)

*At 72 hours:*

- Cumulative doses of noradrenaline, propofol, midazolam, dexmedetomidine
- Cumulative doses of remifentanil, fentanyl, sufentanil, oxycodone, morphine
- Cumulative doses of paracetamol/acetaminophen

## At ICU discharge

- Time of ICU discharge
- Was the patient readmitted to ICU (yes/no)
- Time and results of coronary angiography (1-vessel, 2-vessel, 3-vessel disease)
- Culprit lesion found on coronary angiography (yes/no)
  - Was the culprit lesion an acute thrombotic occlusion (yes/no)
  - Where was the culprit lesion (RCA, Cx, LAD or graft)
- PCI performed (yes/no)
- Coronary artery bypass grafting (CABG) performed (yes/no)
- Patient received an ICD before leaving hospital (yes/no)
- Last left ventricular ejection fraction measurement (normal-hyperdynamic, mildly reduced, moderately reduced, severely reduced)
- Date, time, and value of highest cardiac troponin measurement
- First routine electroencephalogram (EEG) performed (yes/no)
  - Date and time of first routine EEG
  - Highly malignant pattern (burst suppression, suppression, no)
  - Reactive to external stimuli (yes/no)
- Second routine EEG performed (yes/no)
- Continuous EEG performed
  - Highly malignant pattern (burst suppression, suppression, no)
  - Reactive to external stimuli (yes/no)
  - Earliest normal background (hours from randomization)
- Somatosensory evoked potential (SSEP) performed (yes/no)
  - Date and time
  - Indicative of a poor prognosis (yes/no)
- Second SSEP performed during ICU stay (yes/no)
- Computed tomography (CT) brain performed (yes/no)
  - Date and time
  - Indicative of a poor prognosis (yes/no)
  - Bleeding (yes/no)
- Second CT performed during hospital stay (ues/no)
- Magnetic resonance imaging (MRI) of the brain performed (yes/no)
  - Date and time
  - Indicative of a poor prognosis (yes/no)
- Second MRI performed during ICU stay (yes/no)
  - Date and time
  - Indicative of poor prognosis (yes/no)
- Neuron-specific enolase (NSE) measured (yes/no)
  - NSE above 60 ng/ml (or established cutoffs for poor outcome) at 48 or 72 hours (yes/no)
  - NSE concentrations at 24-, 48-, and 72-hours post-arrest
  - Potential confounders malignancies, hemolysis, extracorporal membrane oxygenation (ECMO) other (yes/no)
- Neurofilamen light chain (NFL) locally samples
  - Indicative of a poor prognosis (yes/no)
  - NFL concentrations at 24-, 48-, and 72-hours post-arrest
- Use of mechanical cardiac support and when this was started
  - Impella (or other percutaneous ventricular assist device)
  - ECMO
  - Intra-aortic balloon pump
- Use of haloperidol, olanzapine, quetiapine (yes/no)
- Use of antiseizure medication (yes/no)
- Discharge facility (coronary care unit/general ward/other ICU/dead)
- Use of temperature control device (yes/no/type)
- Continues sedation for the entire 36 hours post randomization (yes/no)
- Time of final extubation (or still intubated)
- Time of awakening
- Highest creatinine during ICU stay
- Renal Replacement Therapy (yes/no)
- Safety events
- Neurological prognostication performed (yes/no)
  - Date and time
  - Confounding factors such as severe metabolic derangement and lingering sedation has been ruled out (yes/no)
  - Hours from the time of last dose of sedative agent to prognostication
  - FOUR motor response at the timepoint of prognostication (makes sign, localising to pain, flexion response to pain, extension response to pain, no response to pain or generalised status myoclonus, or not assessed)
  - Corneal reflexes or pupillary reflexes bilaterally absent at the timepoint of prognostication (yes/no/not assessed)
  - Status myoclonus <72 h post arrest (present, absent, not assessed)
  - Reasons for not performed at 72 hours
- Withdrawal of life-sustaining therapies (yes/no)

## At hospital discharge

- Date and time of hospital discharge as obtained from hospital notes or registries
- Discharged to: nursing home/rehabilitation unit/other hospital/home/dead
- Death (yes/no, time of death)
  - Presumed cause of death (cerebral, cardiac, multi-organ failure, brain death, other)
- Probable cause of cardiac arrest
- Last creatinine
- Patient still on dialysis when discharged from primary hospital (yes/no)
- Additional data on observational parameters (including data such as, but not limited to, circulatory indices, EEG, imaging, pupillometry, near infrared spectroscopy etc.)

## 30 days after randomization

- Date, place (at an institution, in the home of the patient, by telephone, by a digital meeting, other), and current residence (home, hospital, nursing home, rehab facility, other) of follow up,
- If the patient is deceased, date of death, presumed cause of death: cardiac/cerebral/other
- Wake up during the ICU stay (yes/no)
- Wake up before the 30 day follow up (yes/no)
- mRS assessment by telephone interview
- Last and first memory in relation to the cardiac arrest

## 6 months after randomization

- Survival status obtained from hospital or civil registries
- Date of death
- mRS assessment
- EQ-5D-5L VAS

## Biomarker substudy

*Samples will be collected at:*

- 12h after randomization: serum vial 6 ml and plasma vial 6 ml
- 24h after randomization: serum vial 6 ml and plasma vial 6 ml
- 48h after randomization: serum vial 6 ml and plasma vial 6 ml, pax-RNA tube 2.5 ml
- 72h after randomization: serum vial 6 ml and plasma vial 6 ml

| Table 1. Reasons for deviation from the allocated temperature management strategy | |
| --- | --- |
| **Direction of deviation** | **Reason of deviation** |
| Without device | Severe reaction to pharmacological agents |
| Without device | Unsuitable for NSAID (NSAID intolerance, severe asthma, renal failure, renal artery stenosis etc.) |
| Without device | Unsuitable for acetaminophen (liver cirrhosis etc.) |
| Without device | Other reasons |
| With device | Device skin complication (surface device) |
| With device | Device infection (intravascular) |
| With device | Device bleeding/thrombosis (intravascular) |
| With device | Other reasons |
|  | |

| Table 2. Criteria for a likely poor neurological outcome in the STEPCARE trial (according to ERC/ESICM recommendations)^5^ |
| --- |
| In the STEPCARE trial prognosis is considered *likely poor* if criteria A, B and C are all fulfilled: |
| **A.** Confounding factors such as severe metabolic derangement and lingering sedation have been ruled out. The ERC/ESICM recommends awaiting 5 half-lives of the sedative with the longest half-life prior to clinical evaluation. |
| **B.** The patient has no response, a stereotypic extensor response or a stereotypic flexor response to bilateral central and peripheral painful stimulation at ≥ 72 h after randomization. |
| **C.** At least two of the below mentioned signs of a poor prognosis are present:  **C1.** No pupillary AND corneal reflexes ≥72 hours after randomization  **C2.** Bilaterally absent SSEP N20-potentials  **C3.** Early generalized and persisting myoclonus (myoclonic jerks persisting ≥30 min) ≤72 hours after randomization  **C4.** Highly malignant and unreactive EEG-pattern >24 hours after randomization  **C5.** Diffuse and extensive hypoxic brain injury on CT/MRI  **C6.** High NSE >60 ug/L at 48 and/or 72 hours after randomization |

| Table 3. Characteristics of the participants^§^ | **Fever management without a device**  **(N=x)** | **Fever management with a device**  **(N=x)** |
| --- | --- | --- |
| Demographic characteristics |  |  |
| Age ─ yr | x | x |
| Male sex ─ no (%) | x | x |
| Medical history ─ no. (%) |  |  |
| Estimated pre-arrest functional status |  |  |
| Independent in basic activities of life | x | x |
| Dependent in basic activities of life | x | x |
| Percutaneous coronary intervention | x | x |
| Coronary artery bypass grafting | x | x |
| Heart failure with pharmacologic treatment | x | x |
| Implantable cardioverter defibrillator | x | x |
| Hypertension with pharmacologic treatment | x | x |
| Stroke or transitory ischemic attack | x | x |
| Chronic obstructive pulmonary disease | x | x |
| Diabetes mellitus | x | x |
| Kidney disease* | x | x |
| Characteristics of cardiac arrest ─ no. (%) |  |  |
| Scene of cardiac arrest |  |  |
| Home | x | x |
| Work | x | x |
| Public place | x | x |
| Nursing facility | x | x |
| Ambulance | x | x |
| Other | x | x |
| Bystander witnessed cardiac arrest | x | x |
| Bystander performed cardiopulmonary resuscitation | x | x |
| Changing rhythms (any 2 of VF/PEA/asystole) | x | x |
| Adrenaline administered** | x | x |
| Pupillary reflexes present bilaterally prior randomization ─ no./total no. (%) | x | x |
| First monitored rhythm ─ no. (%) |  |  |
| Ventricular fibrillation | x | x |
| Ventricular tachycardia | x | x |
| ROSC after bystander-initiated defibrillation | x | x |
| Unknown shockable rhythm | x | x |
| Pulseless electrical activity | x | x |
| Asystole | x | x |
| Unknown non-shockable rhythm | x | x |
| Median time from cardiac arrest to initiation of advanced life support ─ min (IQR) | x | x |
| Median time from cardiac arrest to ROSC ─ min (IQR) | x | x |
| Median time from ROSC to randomization ─ min (IQR) | x | x |
| Clinical characteristics at hospital admission: |  |  |
| First recorded temperature ─ (◦C) | x | x |
| First pH | x | x |
| Clinical characteristics at ICU admission |  |  |
| FOUR motor score¶ | x | x |
| Corneal reflexes present bilaterally ─ no./total no. (%) |  |  |
| Present bilaterally | x | x |
| Pupillary reflexes present bilaterally ─ no./total no. (%) |  |  |
| Present bilaterally | x | x |
| Circulatory shock# ─ no. (%) | x | x |
| ST-segment elevation myocardial infarction ─ no. (%) | x | x |
| *VF*, ventricular fibrillation, *PEA*, pulseless electrical activity, *IQR* interquartile range, and *ROSC* return of spontaneous circulation.  §Some of these data may be presented in a table/tables in the supplement  *Estimated glomerular filtration rate <30 ml/min/1.73 m^2^  **Prior ROSC and hospital arrival by emergency medical service  ¶Full Outline of Unresponsiveness (FOUR) motor scores range from 0 to 4, with higher scores indicating better motor function  #Shock on admission is defined as a systolic blood pressure of less than 90 mmHg for at least 30 min or the need for supportive measure to maintain a systolic blood pressure ≥ 90 and end organ hypoperfusion (cool extremities, or urine output of less than 30 ml/hr and heart rate >60/min | | |

| Table 4. Intervention-specific information | **Fever management without a device**  **(N=x)** | **Fever management with a device**  **(N=x)** |
| --- | --- | --- |
| Type of device |  |  |
| Invasive device – no. (%) |  | x |
| Non-invasive device – no. (%) |  | x |

| Table 5. Primary and secondary outcomes, and predefined serious adverse events. | | | | |
| --- | --- | --- | --- | --- |
| **Outcome** | **Fever management without a device**  **N =x** | **Fever management with a device**  **N=x** | **Relative risk (95% CI)** | **Absolute risk difference**  **(95% CI)** |
| **Primary outcome** |  |  |  |  |
| 180-day all-cause mortality, no. (%) |  |  |  |  |
| **Secondary outcome** |  |  |  |  |
| mRs 4-6 or dependent on others for basic activities of daily living, no (%) † |  |  |  |  |
| EQ-VAS††, mean (SD) |  |  |  |  |
| Death from any cause in ICU, no. (%) |  |  |  |  |
| **Predefined severe adverse event, no. (%)** |  |  |  |  |
| Sepsis or septic shock^#^ |  |  |  |  |
| Arrhythmia requiring chest compressions or defibrillation |  |  |  |  |
| Moderate or severe bleeding according to Gusto criteria^¶^ |  |  |  |  |
| † mRs denotes modified Rankin scale which ranges from 0 to 6 with 0 indicating no symptoms, 1 symptomatic but not disabled, 2 disabled but independent, 3 dependent but ambulatory, 4 not ambulatory nor capable of body self-care, 5 requires constant care and 6 death  †† EQ VAS denotes EuroQol visual analog scale with 0 indicating worst imaginable health and 100 best imaginable health  # Sepsis III criteria^6^  ¶ Any bleeding requiring blood transfusion, intracerebral bleeding or bleeding resulting in substantial hemodynamic compromise requiring treatment | | | |  |


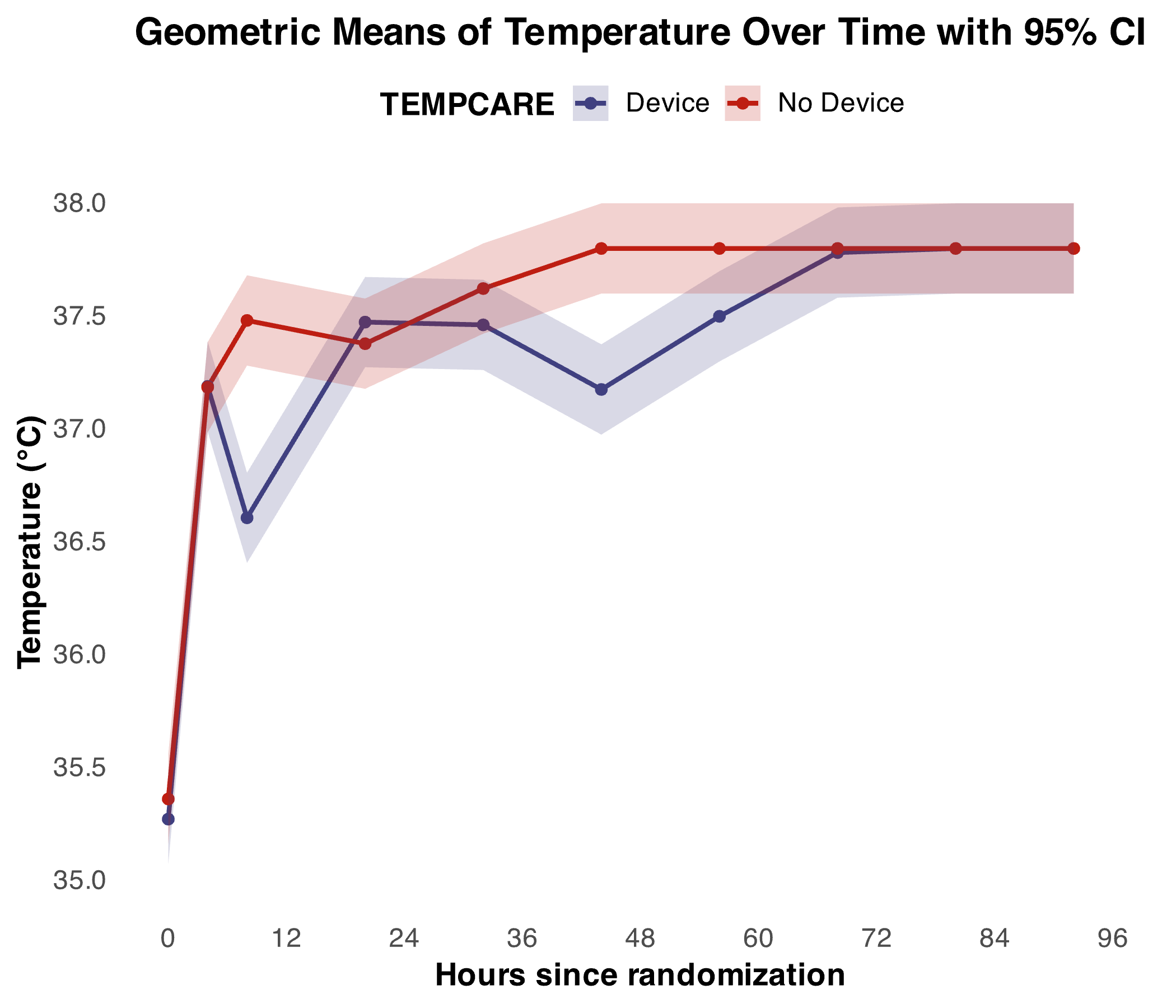


# Figure 1. Mock-figure of geometric means of temperature over time with 95% confidence interval (CI) in the fever management without a device and fever management with a device groups.


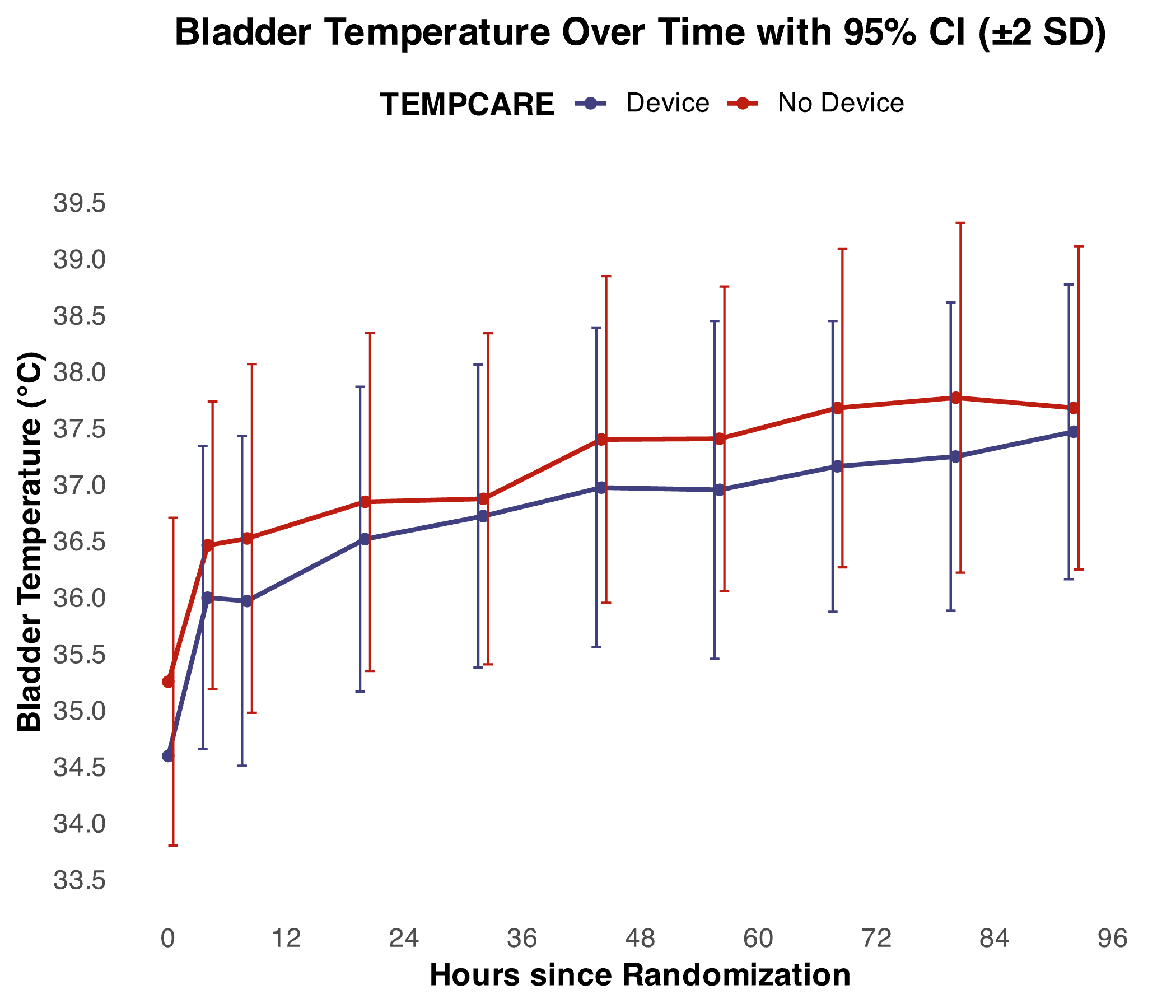


# Figure 2. Mock-figure of mean temperature ± 2 SD in the fever management without a device and fever management with a device groups. Values shown are medians and error bars indicate 2 standard deviations.

# Figure 3. Mock-figure of Kaplan-Meier estimates of the probability of survival until 180 days after randomization in the fever management without a device and fever management with a device groups.

**References:**

1. Nolan JP, Sandroni C, Böttiger BW, et al. European Resuscitation Council and European Society of Intensive Care Medicine guidelines 2021: post-resuscitation care. *Intensive Care Med* 2021; **47**(4): 369-421.

2. Sandroni C, Cariou A, Cavallaro F, et al. Prognostication in comatose survivors of cardiac arrest: an advisory statement from the European Resuscitation Council and the European Society of Intensive Care Medicine. *Intensive Care Med* 2014; **40**(12): 1816-31.

3. Haywood K, Whitehead L, Nadkarni VM, et al. COSCA (Core Outcome Set for Cardiac Arrest) in Adults: An Advisory Statement From the International Liaison Committee on Resuscitation. *Circulation* 2018; **137**(22): e783-e801.

4. Herdman M, Gudex C, Lloyd A, et al. Development and preliminary testing of the new five-level version of EQ-5D (EQ-5D-5L). *Qual Life Res* 2011; **20**(10): 1727-36.

5. Sandroni C, Cariou A, Cavallaro F, et al. Prognostication in comatose survivors of cardiac arrest: an advisory statement from the European Resuscitation Council and the European Society of Intensive Care Medicine. *Resuscitation* 2014; **85**(12): 1779-89.

6. Singer M, Deutschman CS, Seymour CW, et al. The Third International Consensus Definitions for Sepsis and Septic Shock (Sepsis-3). *JAMA* 2016; **315**(8): 801-10.
